# The Wesleyans and Hospital Sunday

**Published:** 1887-01-22

**Authors:** John May

**Affiliations:** Hon. Sec. Ridge Hill, near Macclesfield


### THE WESLEYANS AND HOSPITAL SUNDAY.

Will you do me the favour to inform me whether the Wesleyan ministers of the Metropolis join in supporting the Hospital Sunday collections? My reason for asking is, that here they decline to have collections on Hospital Sunday in behalf of our General Infirmary and Dispensary, including a children's ward, recently opened by the Duchess of Westminster, although every other church and chapel cheerfully assists. I am writing our annual report, and I wish to show what the Wesleyans do in London, Birmingham, and Liverpool.—Yours faithfully, John May, Hon. Sec.

Ridge Hill, near Macclesfield.

*** We have never heard of such a refusal before in any part of the world. The Wesleyans had collections in one hundred and eleven chapels in London on Hospital Sunday, 1886, and paid in £1,019 18*s*. 6*d*., or an average of £9 3*s*. 9*d*. per place of worship.—[Ed. T. H.]

